# The first case report of acute myocardial infarction in young adult caused by scrub typhus

**DOI:** 10.1097/MD.0000000000035271

**Published:** 2023-09-29

**Authors:** Yan Chen, Zhenfeng Guo, Li Wang, Ningchang Cheng, Cheng Wang

**Affiliations:** a Department of Respiratory, BenQ Medical Center, The Affiliated BenQ Hospital of Nanjing Medical University, Nanjing, China; b Department of Cardiovascular, BenQ Medical Center, The Affiliated BenQ Hospital of Nanjing Medical University, Nanjing, China; c Department of Respiratory, Xinglong Community Health Center, Nanjing, China.

**Keywords:** acute myocardial infarction, scrub typhus

## Abstract

**Rationale:**

Scrub typhus is a zoonotic disease caused by *Orientia tsutsugamushi*, a gram-negative intracellular bacterium and endemic in Asia-Pacific area. Acute myocardial infarction after *Oricntia tsutsugamushi* infection was rarely reported, and young adult was not reported.

**Patient conerns:**

A 33-year-old man came to the emergency complained with chest tightness and fever for 4 days.

**Diagonoses:**

After Weil-Felix agglutination test (titer 1:160) twice, scrub typhus was diagnosed.

**Interventions:**

After treating with a hormonotherapy, antibiotic and ventilator-assisted ventilation, his chest tightness was mild lessened with normal breath and body temperature. However, an emergent coronary angiography performed unnormal, then a percutaneous coronary intervention was realized with the implantation of a stent.

**Outcomes:**

After the surgeon, his chest tightness was totally released, and he was discharged.

**Lessons:**

Our case was the first report of young adult acute myocardial infarction after *O tsutsugamushi* infection and we tried to figure out the potential mechanism and how to deal with.

## 1. Introduction

Scrub typhus is a vector-borne zoonosis caused by the organism *Oricntia tsutsugamushi*,^[1]^ and endemic in Asia-Pacific area.^[2,3]^ The most typical symptoms in adults were fever and headache, and fever in pediatric.^[3,4]^ Cardiac manifestations caused by *O tsutsugamushi* infection including angina, bradycardia, and electrocardiogram (ECG) abnormalities were fewer reported recently.^[5–7]^ Here we reported a young man with acute myocardial infarction (AMI) after *O tsutsugamushi* infection and tried to figure out the potential mechanism and how to deal with.

## 2. Case presentation

**Figure 1.**
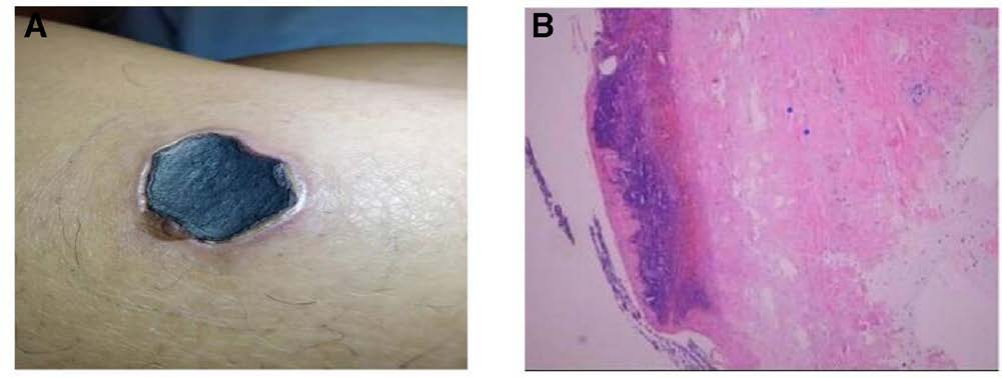
(A) A eschar was seen on the patient’s lower limbs. (B) Pathological examination of skin biopsy specimens of the lower limbs showed mild hyperplasia of surface squamous epithelium and small vessels in the superficial dermis, with focal lymphocyte infiltration, and hyperplasia of collagen fibers in the dermis.

**Figure 2.**
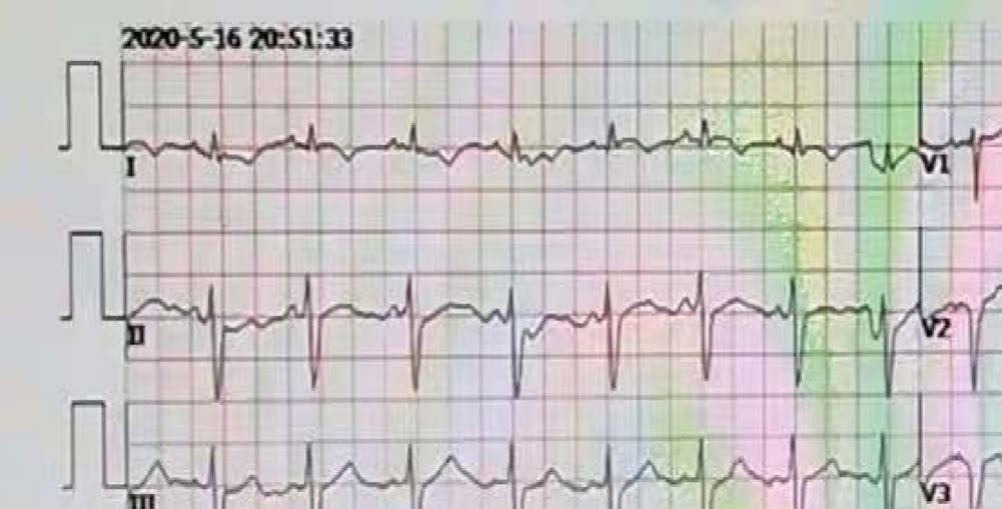
ECG showed sinus tachycardia, shortened P-R interval, intraventricular block, and ST elevation in V2–6, and abnormal Q wave in V2–5. ECG = electrocardiogram.

**Figure 3.**
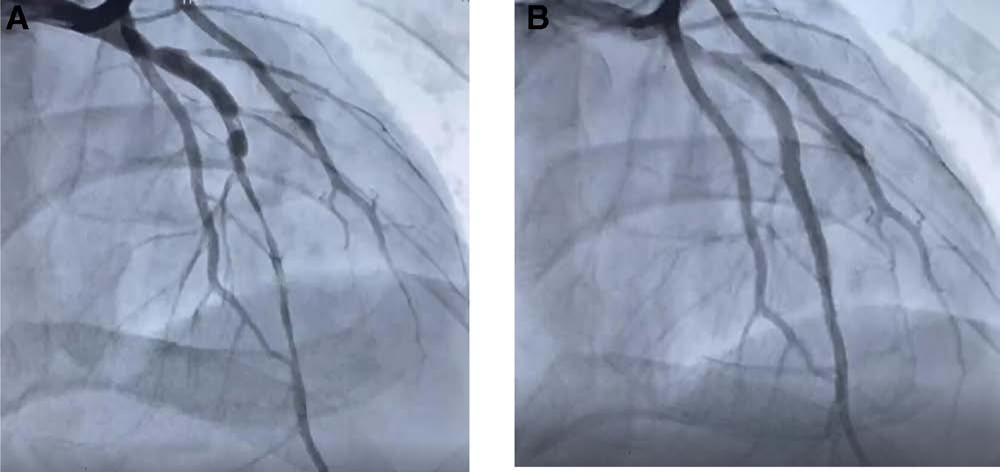
(A) Coronary angiogram demonstrated 50% stenosis in the proximal segment of LAD and 85–90% stenosis in the middle segment, showing a thrombus shadow. (B) Three stents were placed in the near and middle segment of LAD. LAD = left anterior descending artery.

A 33-year-old man came to the emergency on 6th April 2020 with chest tightness and fever (37.5°C–38.8°C) for 4 days. Laboratory data revealed WBC 16.88 × 10^9^/L, CRP 37 mg/L, D-dimer 4.7 mg/L. Chest CT scan showed diffuse patches in bilateral lungs, clearly low lobe. ECG performed negative. He denied hypertension, diabetes, smoking history, and family history of hereditary diseases. After several hours therapy, his chest tightness worsened and dyspnea was present, so he was sent to ICU. At admission, physical examination was performed with rough breath sound with moist rale, and an eschar with 3 to 5 cm diameter sized was found in the right lower limb (Fig. 1). Cardiac enzymes were seriously raised, D-dimer 4.7 mg/L, CK-MB > 300 ng/mL, and troponin T 1166 pg/mL. ECG showed ST elevation in V_2-4_. The patient was treated with cardiopulmonary supports (endotracheal intubation, and mechanical ventilation), anti-infection (Cefazolin sodium i.v., and Vancomycin i.v.) and anticoagulation (low molecular weight heparin s.c.). According to the second time Weil-Felix agglutination test (titer 1:160) positive result, scrub typhus was certainly diagnosed, and after 7 days oral minocycline his chest tightness was mild lessen with normal breath and body temperature. Cardiac enzymes decreased D-dimer 6.56 mg/L, CK-MB 2.1 ng/mL, and TnT 204.1 pg/mL. However, ECG showed sinus tachycardia, ST elevation in V_2-6_, and abnormal Q wave in V_2-5_ (Fig. 2). An emergent coronary angiography was performed with a 50% stenosis in the proximal segment and 85% to 90% stenosis in the middle segment of the left anterior descending artery, and a thrombus can be visible in the middle segment (Fig. 3A). A percutaneous coronary intervention of the left anterior descending artery was realized with the implantation of a stent (Fig. 3B). After percutaneous coronary intervention, his chest tightness was totally released, and he was discharged. The patient has provided informed consent for publication of the case.

## 3. Discussion

*O tsutsugamushi* infection as well as other rickettsial infections would involve several organs including lung, liver, heart, and brain. It was supposed that organ injury results from microvascular dysfunction, which was caused through endothelial cells (ECs) infection and inflammation. Moreover, ECs inflammation was considered as the vital pathogen, and leaded to endothelial and platelet activation that caused platelet adhesion, microvascular thrombosis, and dysfunction, finally multi-organ failure.^[8]^ Recent research revealed furthermore mechanism of ECs malfunction in *O tsutsugamushi* infection.^[9,10]^ Additionally, the immune-mediated host response combined with humoral, and cell mediated immunity also contributed to the pathology. Ge H reported that parts of interleukins, chemokines, and growth factors were highly induced early post *O tsutsugamushi* infection, whereas interferon-α2 was profoundly down-regulated.^[10]^ Wangsanut T reported that *O tsutsugamushi* specifically stabilized *p*105 to inhibit the canonical NF-κB pathway in vitro.^[11]^

Acute bacterial and viral infections were tightly associated with an increased risk of myocardial infarction.^[12]^ However, AMI following *O tsutsugamushi* infection was rarely reported, and firstly reported by South Korea counterpart in 2007.^[13]^ Scrub typhus patients autopsied revealed that most case hearts were pale, soft, flabby, or slightly friable, only 2 case hearts contained small atheromatous plaques in the proximal portions of coronary arteries.^[14]^ Cerebral, splenic infractions caused by *O tsutsugamushi* infection were also reported in recent years.^[15,16]^ So, it was seemed that *O tsutsugamushi* infection would rarely induce large vessel infarction in brain, spleen, and heart, probably involved via EC inflammation or immune-mediated host response. More clinical research was required to figuring out the exact relationship between *O tsutsugamushi* infection and large vessel infarction. In this case, we reported AMI caused by *O tsutsugamushi* infection in a young adult (aged <35) firstly in the world overall. The young man denied hypertension, diabetes, and smoking history, all the high-risk factors of AMI were excluded. Therefore, his AMI was most probably caused by *O tsutsugamushi* infection.

Cardiac injury was commonly seen in patients with *O tsutsugamushi* infection, presenting as NT pro-BNP, troponin T, and CK-MB levels elevated as well as ejection fraction reduced in echocardiography, and ECG abnormalities.^[6,17]^ Myocardial injury, myocarditis, and left ventricular systolic disfunction were frequently recognized and associated with increased morbidity but not mortality.^[18]^ Due to this phenomenon, AMI as a rare cardiac disease in scrub typhus was easily misdiagnosed. According to this spot, we supposed the operation of coronary computed tomography angiography or digital subtraction angiography as early as possible would benefit the patients with cardiac manifestations of *O tsutsugamushi* infection.

*O tsutsugamushi* infection might induce high morbidity and mortality despite low-cost and high-effect antibiotic therapy such as doxycycline, due to delayed recognition and diagnosis. Weil-Felix test first described in 1916, was a test used in the diagnosis of rickettsial infection world widely. However, IgM ELISA became available for scrub typhus diagnosis due to its better specificity and sensitivity.^[19]^ Nowadays, novel diagnosing methods such as IgM immunofluorescence assay (IFA), metagenomic next-generation sequencing (mNGS) were available. IFA was considered as the gold standard diagnostic test instead of IgM ELISA.^[20]^ mNGS performed better than conventional clinical methods to early diagnose scrub typhus.^[21]^ In this case, Weil-Felix test was performed twice without IgM ELISA or IgM IFA, and the patient refused mNGS due to high cost. Although a typical skin change was present in this case, the recognition and diagnosis were delayed due to the conventional diagnosis methods, and novel clinical methods were required for early diagnosis.

## Author contributions

**Conceptualization:** Ningchang Cheng.

**Funding acquisition:** Ningchang Cheng.

**Investigation:** Yan Chen, Ningchang Cheng, Cheng Wang.

**Methodology:** Yan Chen, Ningchang Cheng, Cheng Wang.

**Writing – original draft:** Zhenfeng Guo, Li Wang, Ningchang Cheng.

**Writing – review & editing:** Zhenfeng Guo, Li Wang, Ningchang Cheng.

## References

[1] Rajapakse S Rodrigo C Fernando D. Scrub typhus: pathophysiology, clinical manifestations, and prognosis. Asian Pac J Trop Med. 2012;5:261–4. 22449515 10.1016/S1995-7645(12)60036-4

[2] Panigrahi A Narasimham MV Biswal M. Epidemiology of scrub typhus in a tertiary care hospital of Southern Odisha: a cross-sectional study. Indian J Med Microbiol. 2022;42:92–6. 36192256 10.1016/j.ijmmb.2022.09.005

[3] Kore VB Mahajan SM. Recent threat of scrub typhus in India: a narrative review. Cureus. 2022;14:e30092. 36381766 10.7759/cureus.30092 PMC9641991

[4] Mandal AK Jana JK. 184 Clinical Profile, Laboratory Profile, and Complications of Pediatric Scrub Typhus in Rural Eastern India. BMJ Publishing Group Ltd;2022.

[5] Manappallil RG Nambiar J Anil R. Afebrile scrub typhus infection with cardiac manifestation. BMJ Case Rep. 2021;14:e240223. 10.1136/bcr-2020-240223 PMC8051404 33849868

[6] Choi SW Yun NR Choi D-H. Scrub typhus and abnormal electrocardiography. Am J Trop Med Hyg. 2019;100:399–404. 30734694 10.4269/ajtmh.17-0565 PMC6367634

[7] Jung LY Jeon M Choi SH. Relative bradycardia in scrub typhus. Am J Trop Med Hyg. 2017;97:1316–8. 29016300 10.4269/ajtmh.17-0259 PMC5817766

[8] Gunasekaran K Bal D Varghese GM. Scrub typhus and other rickettsial infections. Indian J Crit Care Med. 2021;25(Suppl 2):S138–43. 34345128 10.5005/jp-journals-10071-23841 PMC8327791

[9] Trent B Liang Y Xing Y. Polarized lung inflammation and Tie2/angiopoietin-mediated endothelial dysfunction during severe Orientia tsutsugamushi infection. PLoS NeglTrop Dis. 2020;14:e0007675. 10.1371/journal.pntd.0007675 PMC7067486 32119672

[10] Ge H Farris CM Tong M. Transcriptional profiles of cytokines and chemokines reveal an important proinflammatory responses of endothelial cells during Orientia tsutsugamushi infection. Microbes Infect. 2019;21:313–20. 30684683 10.1016/j.micinf.2019.01.002

[11] Wangsanut T Brann KR Adcox HE. Orientia tsutsugamushi modulates cellular levels of NF-kappaB inhibitor p105. PLoS NeglTrop Dis. 2021;15:e0009339. 10.1371/journal.pntd.0009339 PMC8078813 33857149

[12] Musher DM Abers MS Corrales-Medina VF. Acute infection and myocardial infarction. N Engl J Med. 2019;380:171–6. 30625066 10.1056/NEJMra1808137

[13] Kim DG Kim JW Choi YS. Acute myocardial infarction following scrub typhus infection. Int J Cardiol. 2007;114:e18–20. 17055080 10.1016/j.ijcard.2006.07.131

[14] Levine HD. Pathologic study of thirty-one cases of scrub typhus fever with especial reference to the cardiovascular system. Am Heart J. 1946;31:314–28. 21018737 10.1016/0002-8703(46)90313-4

[15] Chung JH Yun N-R Kim D-M. Scrub typhus and cerebrovascular injury: a phenomenon of delayed treatment? Am J Trop Med Hyg. 2013;89:119–22. 23716407 10.4269/ajtmh.13-0094 PMC3748467

[16] Tayade A Acharya S Balankhe N. Splenic infarction complicating scrub typhus. J Glob Infect Dis. 2020;12:238–9. 33888970 10.4103/jgid.jgid_27_20 PMC8045541

[17] Pannu AK Debnath MK Sharma N. Circulating cardiac biomarkers and echocardiographic abnormalities in patients with scrub typhus: a prospective cohort study from a tertiary care center in North India. J Vector Borne Dis. 2021;58:193–8. 35170455 10.4103/0972-9062.321754

[18] Karthik G Sudarsan TI Peter JV. Spectrum of cardiac manifestations and its relationship to outcomes in patients admitted with scrub typhus infection. World J Crit Care Med. 2018;7:16–23. 29430404 10.5492/wjccm.v7.i1.16 PMC5797972

[19] Koraluru M Bairy I Varma M. Diagnostic validation of selected serological tests for detecting scrub typhus. Microbiol Immunol. 2015;59:371–4. 26011315 10.1111/1348-0421.12268

[20] Gautam R Parajuli K Tshokey T. Diagnostic evaluation of IgM ELISA and IgM immunofluorescence assay for the diagnosis of acute scrub typhus in central Nepal. BMC infected Dis. 2020;20:138. 10.1186/s12879-020-4861-y PMC7020552 32054525

[21] Liu X Zhang Y Zhang J. The early diagnosis of scrub typhus by metagenomic next-generation sequencing. Front Public Health. 2021;9:755228. 34858931 10.3389/fpubh.2021.755228 PMC8632043

